# The 1:1 adduct of caffeine and 2-(1,3-dioxoisoindolin-2-yl)acetic acid

**DOI:** 10.1107/S1600536811030182

**Published:** 2011-08-02

**Authors:** Moazzam H. Bhatti, Uzma Yunus, Sohail Saeed, Syed Raza Shah, Wing-Tak Wong

**Affiliations:** a Department of Chemistry, Research Complex, Allama Iqbal Open University, Islamabad 44000, Pakistan; b Department of Chemistry, The University of Hong Kong, Pokfulam Road, Pokfulam, Hong Kong SAR, People’s Republic of China

## Abstract

In the crystal structure of the title adduct [systematic name: 2-(1,3-dioxoisoindolin-2-yl)acetic acid–1,3,7-trimethyl-1,2,3,6-tetra­hydro-7*H*-purine-2,6-dione (1/1)], C_8_H_10_N_4_O_2_·C_10_H_7_NO_4_, the components are linked by an O—H⋯N hydrogen-bond and no proton transfer occurs.

## Related literature

For background to *N*-phthaloylglycine and its derivatives, see: Antunes *et al.* (1998 ▶); Barooah *et al.* (2006*a* ▶,*b* ▶); Khan & Ismail (2002 ▶); Shariat & Abdollahi (2004 ▶); Yunus *et al.* (2008 ▶). 
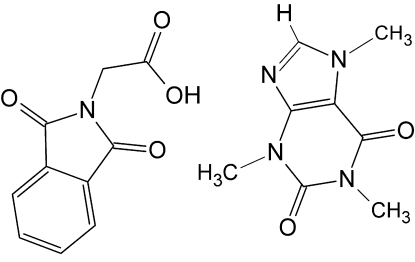


## Experimental

### 

#### Crystal data

C_8_H_10_N_4_O_2_·C_10_H_7_NO_4_
*M* *_r_* = 399.37
Monoclinic, 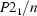

*a* = 14.6595 (5) Å
*b* = 4.6567 (2) Å
*c* = 26.5281 (8) Å
β = 101.408 (2)°
*V* = 1775.16 (11) Å^3^
*Z* = 4
Mo *K*α radiation
μ = 0.12 mm^−1^
*T* = 296 K
0.48 × 0.16 × 0.10 mm

#### Data collection

Bruker APEXII CCD diffractometer
Absorption correction: multi-scan (*SADABS*; Sheldrick, 2008*b* ▶) *T*_min_ = 0.947, *T*_max_ = 0.989
20916 measured reflections
3373 independent reflections
2268 reflections with *I* > 2σ(*I*)
*R*_int_ = 0.045

#### Refinement

*R*[*F* ^2^ > 2σ(*F* ^2^)] = 0.044
*wR*(*F* ^2^) = 0.130
*S* = 1.03
3373 reflections
269 parameters
H atoms treated by a mixture of independent and constrained refinement
Δρ_max_ = 0.20 e Å^−3^
Δρ_min_ = −0.21 e Å^−3^

### 

Data collection: *APEX2* (Bruker, 2007 ▶); cell refinement: *APEX2*; data reduction: *SAINT* (Bruker, 2007 ▶); program(s) used to solve structure: *SHELXS97* (Sheldrick, 2008*a* ▶); program(s) used to refine structure: *SHELXL97* (Sheldrick, 2008*a* ▶); molecular graphics: *Mercury* (Macrae *et al.*, 2008 ▶); software used to prepare material for publication: *SHELXL97*.

## Supplementary Material

Crystal structure: contains datablock(s) global, I. DOI: 10.1107/S1600536811030182/ds2126sup1.cif

Structure factors: contains datablock(s) I. DOI: 10.1107/S1600536811030182/ds2126Isup2.hkl

Supplementary material file. DOI: 10.1107/S1600536811030182/ds2126Isup3.cml

Additional supplementary materials:  crystallographic information; 3D view; checkCIF report

## Figures and Tables

**Table 1:** Hydrogen-bond geometry (Å, °)

*D*—H⋯*A*	*D*—H	H⋯*A*	*D*⋯*A*	*D*—H⋯*A*
O1—H1⋯N5	0.98 (3)	1.73 (3)	2.707 (2)	171 (3)

**Fig. 1.**
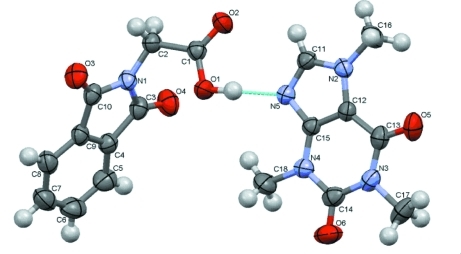
The ORTEP plot of the co-crystal was shown at 50% probability thermal ellipsoids with the atom numbering scheme.

## supplementary crystallographic information

### Comment

Among the *N*-phthaloylamino acids, N-phthaloylglycine is the most widely studied for cleavage with various amines (Khan & Ismail, 2002), metal complexes with interesting supramolecular structures (Barooah *et al.*, 2006*a*) and adduct formation with various aromatic amines and hydroxyl aromatics (Barooah *et al.*, 2006*b*). The heterocyclic derivatives are also known in the literature such as oxadizaole (Antunes *et al.*, 1998), benzoxazinone (Shariat & Abdollahi, 2004) and 1,2,4-triazole (Yunus *et al.*, 2008).

In an attempt to synthesis calcium(II) complex of N-phthaloylglycine and caffeine, we have obtained 1:1 adduct of N-phthaloylglycine and caffeine as title compound (I). The 1,3,7-trimethyl-hexahydro-purine-2,6-dione, C_8_H_10_N_4_O_2_, and (1,3-dioxo-1,3-dihydro-isoindol-2-yl)-acetic acid, C_10_H_7_NO_4_, co-crystallizes in a primitive monoclinic space group, *P*2_1_/*n* (#14). The acetic acid group is about 74.96 (5)° from the ring plane of isoindole-1,3-dione.

C—O distances [C1—O2 = 1.200 (2); C1—O1 = 1.316 (2) Å) of the COOH moiety suggests that no proton transfer has taken place. There are inter-molecular O—H···N H-bond interactions which link the two molecules together.

### Experimental

A mixture of CaCO_3_ (0.005 mol), N-phthaloylglycine (0.01 mol) and caffeine (0.005 mol) was heated in water (100 ml) for 2 h. The hot solution was filtered and filtrate was set aside for one week. Colourless needle like crystals were obtained suitable for X-ray analysis.

### Refinement

The structure was solved by direct methods (*SHELXS97*) and expanded using Fourier techniques. All non-H atoms were refined anisotropically.

All of the C-bound H atoms are observable from difference Fourier map but are all placed at geometrical positions with C—H = 0.93, 0.96 and 0.97 Å for phenyl methyl and methylene H-atoms. All C-bound H-atoms are refined using riding model with *U*_iso_(H) = 1.2*U*_eq_(Carrier). The O-bound H-atoms were located from difference Fourier map and refined isotropically.

Highest peak is 0.20 at (0.2868, 0.9541, 0.0373) [0.97Å from H18A] Deepest hole is -0.21 at (0.9986, 0.7170, 0.1361) [1.03Å from N1]

### Crystal data

**Table:**

C_8_H_10_N_4_O_2_·C_10_H_7_NO_4_	*F*(000) = 832
*M**_r_* = 399.37	*D*_x_ = 1.494 Mg m^−^^3^
Monoclinic, *P*2_1_/*n*	Mo *K*α radiation, λ = 0.71073 Å
Hall symbol: -P 2yn	Cell parameters from 2850 reflections
*a* = 14.6595 (5) Å	θ = 2.9–23.0°
*b* = 4.6567 (2) Å	µ = 0.12 mm^−^^1^
*c* = 26.5281 (8) Å	*T* = 296 K
β = 101.408 (2)°	Needle, colourless
*V* = 1775.16 (11) Å^3^	0.48 × 0.16 × 0.10 mm
*Z* = 4	

### Data collection

**Table:**

Bruker APEXII CCD diffractometer	3373 independent reflections
Radiation source: fine-focus sealed tube	2268 reflections with *I* > 2σ(*I*)
graphite	*R*_int_ = 0.045
ω and φ scan	θ_max_ = 25.7°, θ_min_ = 1.6°
Absorption correction: multi-scan (*SADABS*; Sheldrick, 2008*b*)	*h* = −17→17
*T*_min_ = 0.947, *T*_max_ = 0.989	*k* = −5→5
20916 measured reflections	*l* = −32→32

### Refinement

**Table:**

Refinement on *F*^2^	Primary atom site location: structure-invariant direct methods
Least-squares matrix: full	Secondary atom site location: difference Fourier map
*R*[*F*^2^ > 2σ(*F*^2^)] = 0.044	Hydrogen site location: inferred from neighbouring sites
*wR*(*F*^2^) = 0.130	H atoms treated by a mixture of independent and constrained refinement
*S* = 1.03	*w* = 1/[σ^2^(*F*_o_^2^) + (0.0683*P*)^2^ + 0.2661*P*] where *P* = (*F*_o_^2^ + 2*F*_c_^2^)/3
3373 reflections	(Δ/σ)_max_ = 0.005
269 parameters	Δρ_max_ = 0.20 e Å^−^^3^
0 restraints	Δρ_min_ = −0.21 e Å^−^^3^

### Special details

**Table:**

Geometry. All e.s.d.'s (except the e.s.d. in the dihedral angle between two l.s. planes) are estimated using the full covariance matrix. The cell e.s.d.'s are taken into account individually in the estimation of e.s.d.'s in distances, angles and torsion angles; correlations between e.s.d.'s in cell parameters are only used when they are defined by crystal symmetry. An approximate (isotropic) treatment of cell e.s.d.'s is used for estimating e.s.d.'s involving l.s. planes.
Refinement. Refinement of *F*^2^ against ALL reflections. The weighted *R*-factor *wR* and goodness of fit *S* are based on *F*^2^, conventional *R*-factors *R* are based on *F*, with *F* set to zero for negative *F*^2^. The threshold expression of *F*^2^ > σ(*F*^2^) is used only for calculating *R*-factors(gt) *etc*. and is not relevant to the choice of reflections for refinement. *R*-factors based on *F*^2^ are statistically about twice as large as those based on *F*, and *R*- factors based on ALL data will be even larger.

### Fractional atomic coordinates and isotropic or equivalent isotropic displacement parameters (Å^2^)

**Table:**

	*x*	*y*	*z*	*U*_iso_*/*U*_eq_	
O1	0.22787 (11)	0.5983 (3)	0.16097 (6)	0.0524 (4)	
H1	0.293 (2)	0.537 (6)	0.1631 (11)	0.096 (9)*	
O2	0.29779 (10)	0.9442 (3)	0.21235 (6)	0.0574 (4)	
O3	−0.01236 (11)	0.5371 (4)	0.21311 (6)	0.0592 (4)	
O4	0.08996 (11)	1.0227 (4)	0.08551 (6)	0.0630 (5)	
O5	0.68437 (11)	0.0115 (4)	0.15118 (7)	0.0657 (5)	
O6	0.42324 (13)	−0.2400 (4)	0.03383 (6)	0.0715 (5)	
N1	0.05672 (10)	0.8102 (4)	0.15825 (6)	0.0416 (4)	
N2	0.55660 (11)	0.4293 (4)	0.19480 (6)	0.0420 (4)	
N3	0.55304 (13)	−0.1112 (4)	0.09235 (7)	0.0489 (5)	
N4	0.40577 (12)	0.0946 (4)	0.09313 (6)	0.0440 (4)	
N5	0.40629 (11)	0.4590 (4)	0.15872 (6)	0.0409 (4)	
C1	0.22844 (14)	0.8265 (5)	0.19031 (8)	0.0408 (5)	
C2	0.13310 (13)	0.9294 (5)	0.19542 (8)	0.0459 (5)	
H2A	0.1237	0.8825	0.2297	0.055*	
H2B	0.1313	1.1369	0.1922	0.055*	
C3	0.04313 (14)	0.8602 (5)	0.10534 (8)	0.0451 (5)	
C4	−0.03699 (14)	0.6796 (5)	0.08152 (8)	0.0430 (5)	
C5	−0.07858 (17)	0.6416 (6)	0.03054 (9)	0.0577 (6)	
H5	−0.0582	0.7406	0.0044	0.069*	
C6	−0.15203 (19)	0.4493 (6)	0.02013 (10)	0.0683 (8)	
H6	−0.1806	0.4149	−0.0139	0.082*	
C7	−0.18374 (17)	0.3079 (6)	0.05892 (11)	0.0683 (7)	
H7	−0.2340	0.1831	0.0505	0.082*	
C8	−0.14258 (15)	0.3469 (5)	0.11024 (10)	0.0568 (6)	
H8	−0.1640	0.2516	0.1364	0.068*	
C9	−0.06796 (13)	0.5348 (5)	0.12049 (8)	0.0434 (5)	
C10	−0.00813 (14)	0.6162 (5)	0.17002 (8)	0.0425 (5)	
C11	0.47493 (14)	0.5566 (5)	0.19502 (8)	0.0420 (5)	
H11	0.4670	0.6994	0.2183	0.050*	
C12	0.54018 (13)	0.2291 (4)	0.15529 (7)	0.0389 (5)	
C13	0.60057 (15)	0.0424 (5)	0.13510 (9)	0.0466 (6)	
C14	0.45764 (16)	−0.0942 (5)	0.07064 (8)	0.0488 (6)	
C15	0.44793 (13)	0.2549 (4)	0.13435 (7)	0.0372 (5)	
C16	0.64351 (16)	0.4833 (6)	0.23127 (9)	0.0610 (7)	
H16A	0.6928	0.5142	0.2128	0.073*	
H16B	0.6582	0.3206	0.2536	0.073*	
H16C	0.6366	0.6506	0.2513	0.073*	
C17	0.60798 (19)	−0.3018 (6)	0.06585 (10)	0.0692 (8)	
H17A	0.6630	−0.3617	0.0895	0.083*	
H17B	0.6253	−0.2010	0.0376	0.083*	
H17C	0.5715	−0.4673	0.0531	0.083*	
C18	0.30650 (16)	0.1200 (6)	0.07221 (10)	0.0652 (7)	
H18A	0.2970	0.1605	0.0361	0.078*	
H18B	0.2810	0.2732	0.0893	0.078*	
H18C	0.2761	−0.0569	0.0775	0.078*	

### Atomic displacement parameters (Å^2^)

**Table:**

	*U*^11^	*U*^22^	*U*^33^	*U*^12^	*U*^13^	*U*^23^
O1	0.0379 (9)	0.0535 (10)	0.0644 (10)	0.0043 (7)	0.0064 (7)	−0.0183 (8)
O2	0.0371 (9)	0.0605 (10)	0.0714 (11)	−0.0044 (8)	0.0033 (8)	−0.0154 (8)
O3	0.0578 (10)	0.0744 (11)	0.0468 (9)	0.0017 (9)	0.0134 (8)	0.0143 (8)
O4	0.0560 (10)	0.0731 (11)	0.0613 (11)	−0.0102 (9)	0.0155 (8)	0.0140 (9)
O5	0.0414 (10)	0.0730 (12)	0.0859 (13)	0.0115 (8)	0.0208 (9)	0.0056 (9)
O6	0.0895 (13)	0.0668 (12)	0.0590 (11)	−0.0085 (10)	0.0171 (9)	−0.0233 (10)
N1	0.0319 (9)	0.0526 (10)	0.0395 (10)	0.0017 (8)	0.0047 (7)	0.0017 (8)
N2	0.0363 (9)	0.0465 (10)	0.0430 (10)	−0.0046 (8)	0.0077 (8)	0.0009 (8)
N3	0.0544 (12)	0.0428 (10)	0.0551 (12)	0.0053 (9)	0.0244 (9)	−0.0002 (9)
N4	0.0429 (10)	0.0444 (10)	0.0441 (10)	−0.0007 (8)	0.0071 (8)	−0.0040 (9)
N5	0.0370 (9)	0.0433 (10)	0.0431 (10)	0.0023 (8)	0.0095 (8)	−0.0031 (8)
C1	0.0369 (11)	0.0441 (12)	0.0404 (12)	0.0010 (10)	0.0054 (9)	0.0014 (10)
C2	0.0374 (11)	0.0496 (13)	0.0489 (13)	0.0055 (10)	0.0043 (10)	−0.0073 (10)
C3	0.0381 (11)	0.0517 (13)	0.0464 (13)	0.0073 (10)	0.0105 (10)	0.0073 (11)
C4	0.0374 (11)	0.0473 (12)	0.0435 (12)	0.0077 (10)	0.0058 (9)	−0.0001 (10)
C5	0.0557 (14)	0.0676 (16)	0.0465 (14)	0.0128 (13)	0.0020 (11)	−0.0001 (12)
C6	0.0584 (16)	0.0771 (18)	0.0600 (17)	0.0108 (15)	−0.0113 (13)	−0.0173 (15)
C7	0.0463 (14)	0.0640 (17)	0.089 (2)	−0.0028 (13)	0.0006 (14)	−0.0178 (16)
C8	0.0416 (13)	0.0570 (15)	0.0723 (17)	−0.0018 (11)	0.0123 (12)	−0.0043 (13)
C9	0.0317 (11)	0.0460 (12)	0.0520 (13)	0.0060 (9)	0.0075 (10)	−0.0005 (10)
C10	0.0360 (11)	0.0482 (12)	0.0437 (13)	0.0096 (10)	0.0092 (9)	0.0049 (10)
C11	0.0456 (12)	0.0429 (12)	0.0396 (12)	−0.0009 (10)	0.0132 (10)	−0.0028 (10)
C12	0.0357 (11)	0.0398 (11)	0.0428 (12)	0.0008 (9)	0.0120 (9)	0.0016 (10)
C13	0.0422 (13)	0.0466 (13)	0.0544 (14)	0.0034 (10)	0.0180 (11)	0.0101 (11)
C14	0.0604 (15)	0.0434 (13)	0.0456 (13)	−0.0026 (11)	0.0178 (11)	−0.0010 (11)
C15	0.0382 (11)	0.0367 (11)	0.0382 (11)	−0.0005 (9)	0.0112 (9)	0.0003 (9)
C16	0.0437 (13)	0.0752 (17)	0.0599 (15)	−0.0102 (12)	−0.0005 (11)	−0.0039 (13)
C17	0.0885 (19)	0.0551 (15)	0.0776 (18)	0.0166 (14)	0.0492 (15)	0.0015 (14)
C18	0.0476 (14)	0.0845 (19)	0.0581 (15)	−0.0007 (14)	−0.0024 (11)	−0.0162 (14)

### Geometric parameters (Å, °)

**Table:**

O1—C1	1.316 (2)	C3—C4	1.481 (3)
O1—H1	0.98 (3)	C4—C5	1.380 (3)
O2—C1	1.200 (2)	C4—C9	1.384 (3)
O3—C10	1.214 (2)	C5—C6	1.386 (4)
O4—C3	1.210 (2)	C5—H5	0.9300
O5—C13	1.227 (3)	C6—C7	1.378 (4)
O6—C14	1.214 (3)	C6—H6	0.9300
N1—C10	1.391 (3)	C7—C8	1.388 (3)
N1—C3	1.398 (3)	C7—H7	0.9300
N1—C2	1.448 (2)	C8—C9	1.385 (3)
N2—C11	1.337 (3)	C8—H8	0.9300
N2—C12	1.388 (3)	C9—C10	1.478 (3)
N2—C16	1.461 (3)	C11—H11	0.9300
N3—C13	1.404 (3)	C12—C15	1.362 (3)
N3—C14	1.405 (3)	C12—C13	1.419 (3)
N3—C17	1.468 (3)	C16—H16A	0.9600
N4—C15	1.367 (2)	C16—H16B	0.9600
N4—C14	1.374 (3)	C16—H16C	0.9600
N4—C18	1.456 (3)	C17—H17A	0.9600
N5—C11	1.328 (3)	C17—H17B	0.9600
N5—C15	1.360 (2)	C17—H17C	0.9600
C1—C2	1.509 (3)	C18—H18A	0.9600
C2—H2A	0.9700	C18—H18B	0.9600
C2—H2B	0.9700	C18—H18C	0.9600
			
C1—O1—H1	107.9 (17)	C7—C8—H8	121.7
C10—N1—C3	111.45 (17)	C4—C9—C8	121.6 (2)
C10—N1—C2	124.52 (17)	C4—C9—C10	108.32 (18)
C3—N1—C2	123.87 (17)	C8—C9—C10	130.1 (2)
C11—N2—C12	106.23 (16)	O3—C10—N1	125.0 (2)
C11—N2—C16	125.80 (19)	O3—C10—C9	128.9 (2)
C12—N2—C16	127.89 (18)	N1—C10—C9	106.19 (17)
C13—N3—C14	126.66 (18)	N5—C11—N2	113.31 (18)
C13—N3—C17	117.6 (2)	N5—C11—H11	123.3
C14—N3—C17	115.7 (2)	N2—C11—H11	123.3
C15—N4—C14	119.71 (18)	C15—C12—N2	105.00 (17)
C15—N4—C18	121.35 (18)	C15—C12—C13	122.92 (19)
C14—N4—C18	118.94 (19)	N2—C12—C13	131.95 (19)
C11—N5—C15	103.71 (16)	O5—C13—N3	121.6 (2)
O2—C1—O1	124.22 (19)	O5—C13—C12	126.7 (2)
O2—C1—C2	121.44 (19)	N3—C13—C12	111.71 (18)
O1—C1—C2	114.33 (18)	O6—C14—N4	122.0 (2)
N1—C2—C1	114.98 (17)	O6—C14—N3	121.3 (2)
N1—C2—H2A	108.5	N4—C14—N3	116.71 (19)
C1—C2—H2A	108.5	N5—C15—C12	111.75 (17)
N1—C2—H2B	108.5	N5—C15—N4	125.98 (18)
C1—C2—H2B	108.5	C12—C15—N4	122.25 (18)
H2A—C2—H2B	107.5	N2—C16—H16A	109.5
O4—C3—N1	124.1 (2)	N2—C16—H16B	109.5
O4—C3—C4	129.8 (2)	H16A—C16—H16B	109.5
N1—C3—C4	106.10 (17)	N2—C16—H16C	109.5
C5—C4—C9	121.5 (2)	H16A—C16—H16C	109.5
C5—C4—C3	130.5 (2)	H16B—C16—H16C	109.5
C9—C4—C3	107.92 (17)	N3—C17—H17A	109.5
C4—C5—C6	116.9 (2)	N3—C17—H17B	109.5
C4—C5—H5	121.5	H17A—C17—H17B	109.5
C6—C5—H5	121.5	N3—C17—H17C	109.5
C7—C6—C5	121.6 (2)	H17A—C17—H17C	109.5
C7—C6—H6	119.2	H17B—C17—H17C	109.5
C5—C6—H6	119.2	N4—C18—H18A	109.5
C6—C7—C8	121.7 (2)	N4—C18—H18B	109.5
C6—C7—H7	119.2	H18A—C18—H18B	109.5
C8—C7—H7	119.2	N4—C18—H18C	109.5
C9—C8—C7	116.6 (2)	H18A—C18—H18C	109.5
C9—C8—H8	121.7	H18B—C18—H18C	109.5

### Hydrogen-bond geometry (Å, °)

**Table:**

*D*—H···*A*	*D*—H	H···*A*	*D*···*A*	*D*—H···*A*
O1—H1···N5	0.98 (3)	1.73 (3)	2.707 (2)	171 (3)
